# Osteochondritis Dissecans of the Medial Femoral Condyle With Intra‐Articular Meniscal Calcification: A Rare Case and Literature Review

**DOI:** 10.1155/cro/8357578

**Published:** 2026-03-31

**Authors:** Ekrem Özdemir, Nasuhi Altay

**Affiliations:** ^1^ Department of Orthopedics and Traumatology, Erzurum City Hospital, Erzurum, Türkiye

**Keywords:** arthroscopy, knee joint, loose body, medial femoral condyle, meniscal calcification, microfracture, osteochondritis dissecans

## Abstract

Osteochondritis dissecans (OCD) is a joint disorder characterized by subchondral bone necrosis and potential fragment detachment; although intra‐articular loose bodies are common, a meniscal‐fixed calcified body associated with OCD is exceedingly rare. This case reports a 56‐year‐old male with a Stage 4B OCD lesion on the medial femoral condyle and a concomitant calcified body fixed to the posterior horn of the medial meniscus, which was identified via MRI as a primary driver of chronic mechanical stress and lesion progression. Following arthroscopic excision and microfracture, the patient demonstrated significant clinical recovery at 6 months, with VAS scores decreasing from 7 to 3 and KOOS improving from 15 to 75. This rare association suggests that localized mechanical impingement from meniscal‐fixed calcifications may play a critical role in OCD pathogenesis, emphasizing the necessity of comprehensive imaging to identify such contributing factors for optimized surgical outcomes.

## 1. Introduction

Osteochondritis dissecans (OCD) is a joint disorder characterized by focal subchondral osteochondral lesions that may lead to impaired ossification, transitional discontinuity at the osteochondral junction, and eventual subchondral bone involvement, which may lead to detachment of a fragment of cartilage and bone within the joint space [1]. The knee is the most commonly affected joint, particularly the lateral aspect of the medial femoral condyle, and it predominantly occurs in adolescents and young adults engaged in sports activities [2, 3]. Although the exact etiology of OCD remains unclear, repetitive microtrauma, vascular insufficiency, genetic predisposition, and biomechanical factors have been implicated in its pathogenesis [4].

Intra‐articular loose bodies are known to cause mechanical symptoms such as joint locking, pain, and restricted range of motion. These loose bodies may result from trauma, osteochondral fractures, synovial chondromatosis, or degenerative joint diseases [5]. Meniscal calcified loose bodies, however, are extremely rare and typically arise due to chronic degeneration, trauma, or calcific deposition diseases such as calcium pyrophosphate dihydrate (CPPD) deposition [6].

The presence of a calcified loose body within the knee joint can exacerbate cartilage damage by causing continuous mechanical irritation. Although the association between intra‐articular loose bodies and secondary osteochondral lesions has been previously documented, reports linking meniscal‐origin calcified loose bodies to the development of OCD are exceedingly rare [7].

Herein, we present a rare case of OCD of the medial femoral condyle associated with a meniscal calcified loose body in the knee joint. To the best of our knowledge, this is among the very few cases reported in the literature, highlighting the potential role of mechanical factors in the pathogenesis of OCD.

## 2. Case Presentation

### 2.1. Patient Information

A 56‐year‐old male patient presented with a 6‐month history of right knee pain, intermittent joint locking, and swelling. The patient denied any history of major trauma but reported worsening symptoms after prolonged standing, walking, and stair climbing. His medical history was unremarkable for metabolic, rheumatologic, or degenerative joint diseases.

### 2.2. Clinical Findings

On physical examination, mild joint effusion and tenderness over the medial joint line were noted. The range of motion was limited to 0°–110°, with pain and mechanical blockage observed during terminal flexion. There was no varus or valgus instability on stress testing. Lachman, anterior drawer, and posterior drawer tests were negative. McMurray′s test elicited medial joint line pain without an audible click. The patellar grind test was negative. Gait analysis revealed a mild antalgic gait due to pain. Neurovascular examination of the right lower extremity was normal, with palpable distal pulses, normal capillary refill, and intact motor and sensory function. No muscle atrophy or signs of inflammation were observed.

### 2.3. Timeline

**Figure 1:**
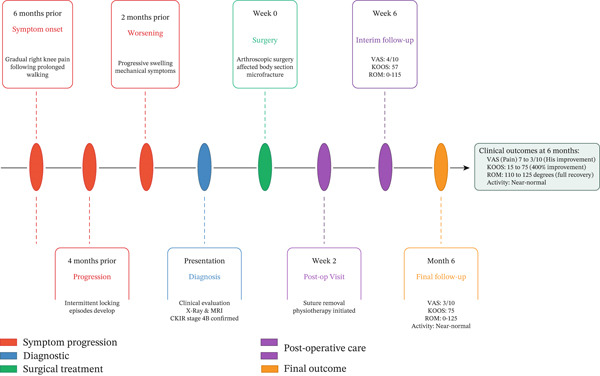
Clinical timeline illustrating the patient′s course from symptom onset to 6‐month follow‐up, including key clinical events, diagnostic procedures, surgical intervention, and outcome measures (VAS, KOOS, and ROM) at each timepoint.

The clinical course, summarized in Figure 1, began 6 months prior to presentation with the gradual onset of right knee pain following prolonged walking, which progressed to intermittent locking episodes at 4 months and worsening swelling with mechanical symptoms by 2 months before the initial evaluation. Following clinical assessment and imaging via plain radiography and magnetic resonance imaging (MRI), the patient underwent arthroscopic excision of the calcified body and microfracture at Week 0, followed by a 2‐week postoperative visit for suture removal and the initiation of physiotherapy. Subsequent follow‐ups demonstrated steady recovery, with an interim assessment at Week 6 showing a visual analog scale (VAS) of 4/10, a Knee injury and Osteoarthritis Outcome Score (KOOS) of 52, and a ROM of 0°–115°, ultimately reaching a final follow‐up at Month 6 with significant clinical improvement, characterized by a VAS of 3/10, a KOOS of 75, and a ROM of 0°–125°.

### 2.4. Diagnostic Assessment

Plain radiographs of the knee (anteroposterior and lateral views) revealed joint space narrowing in the medial compartment and subchondral sclerosis, with subtle irregularity of the medial femoral condyle articular surface (Figure 2). MRI demonstrated a 4B stage OCD lesion measuring approximately 1.5 cm in diameter on the lateral aspect of the medial femoral condyle, consistent with an unstable osteochondral defect (Figure 3). The lesion exhibited low signal intensity on T1‐weighted sequences and heterogeneous signal intensity on T2‐weighted sequences, with surrounding subchondral bone marrow edema indicating lesion instability (Figure 4). Additionally, an irregularly contoured calcified body, approximately 12 mm in diameter, was identified extending towards the posterior horn of the medial meniscus and firmly attached to it. This calcified body was located in the projection area of the OCD lesion. No significant cartilage degeneration or advanced osteoarthritic changes were observed.

Figure 2(a) Anteroposterior (AP) knee radiograph demonstrating joint space narrowing and subchondral sclerosis in the medial compartment, with subtle irregularity of the medial femoral condyle articular surface. (b) Lateral knee radiograph showing subchondral sclerosis and joint space narrowing projecting in the region of the medial compartment.

**Figure:**
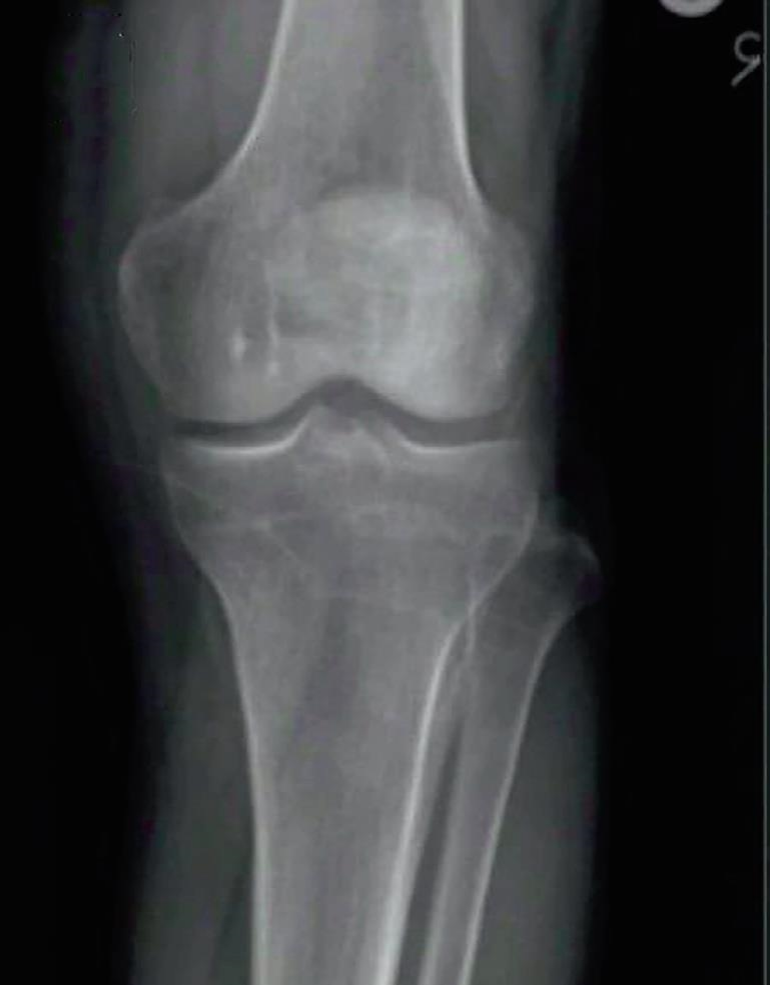
(a)

**Figure:**
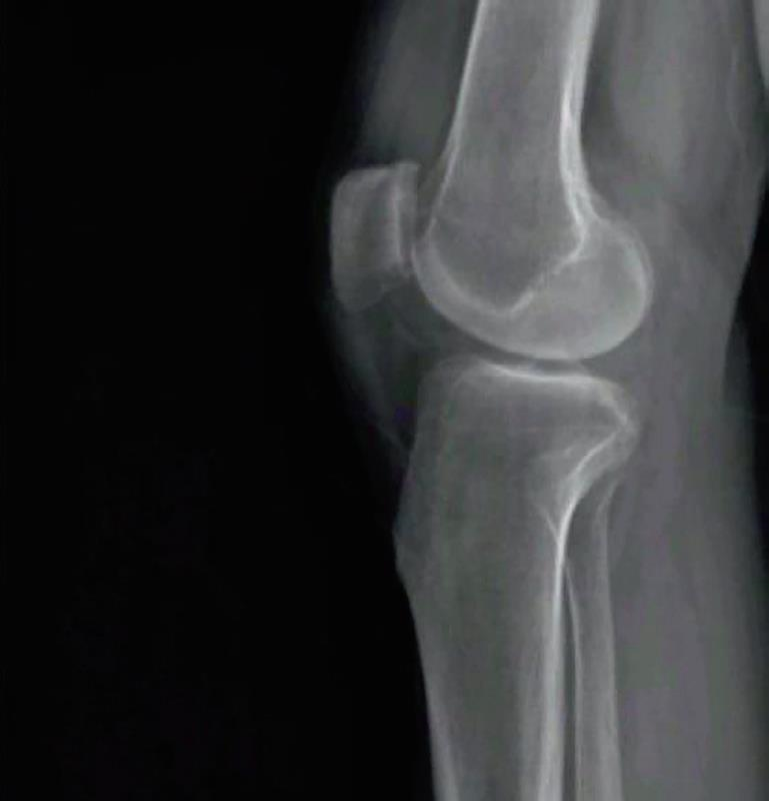
(b)

Figure 3(a) Sagittal MRI image demonstrates a meniscal ossicle. (b) Axial MRI image demonstrates a meniscal ossicle.

**Figure:**
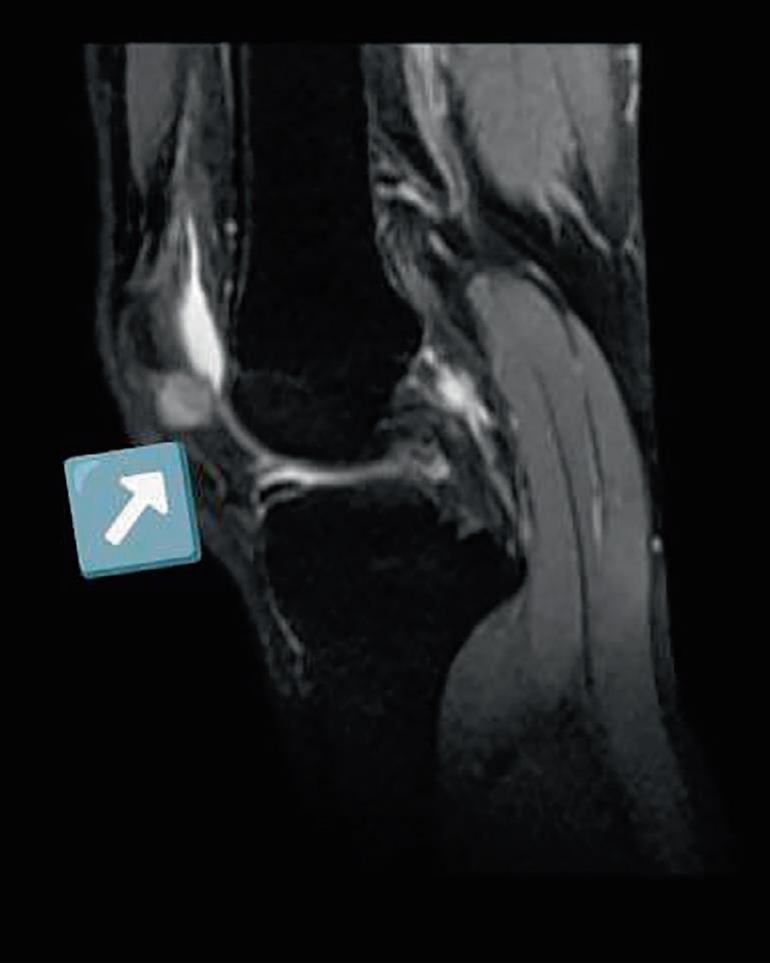
(a)

**Figure:**
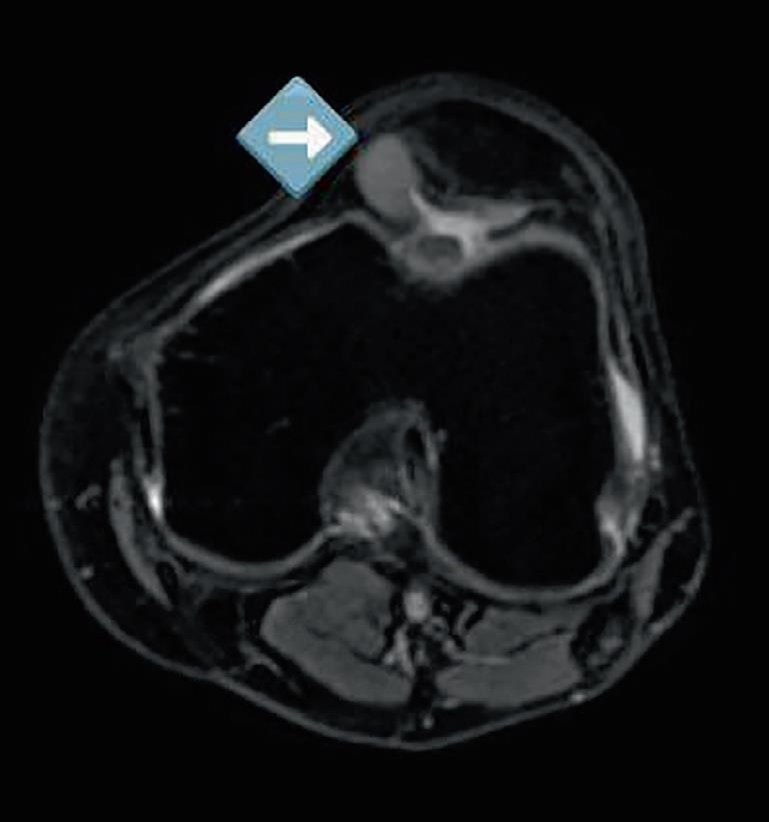
(b)

Figure 4(a) Coronal MRI view demonstrating the osteochondritis dissecans lesion on the medial femoral condyle with low signal intensity on T1‐weighted sequence and surrounding bone marrow edema. (b) Axial MRI view showing the OCD lesion with heterogeneous signal intensity and subchondral changes consistent with lesion instability.

**Figure:**
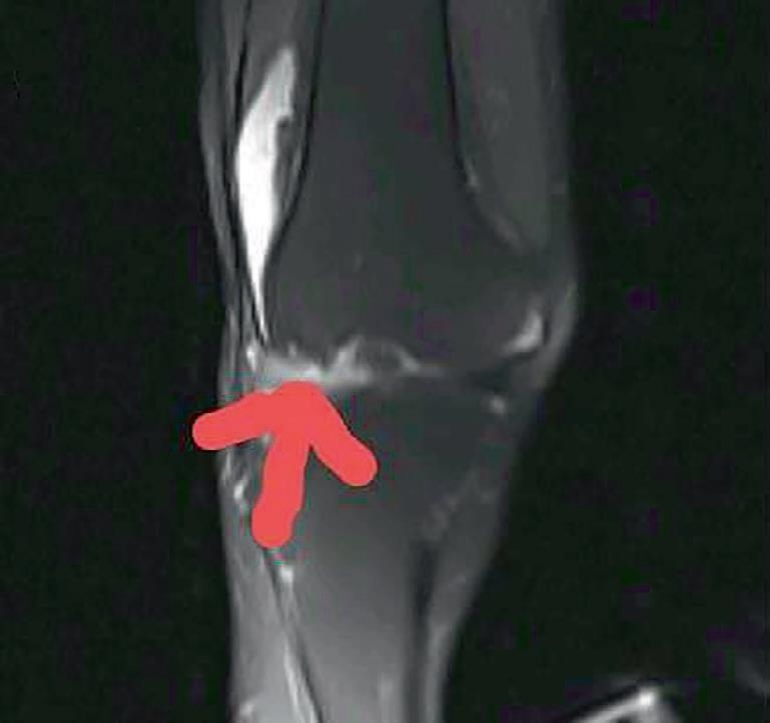
(a)

**Figure:**
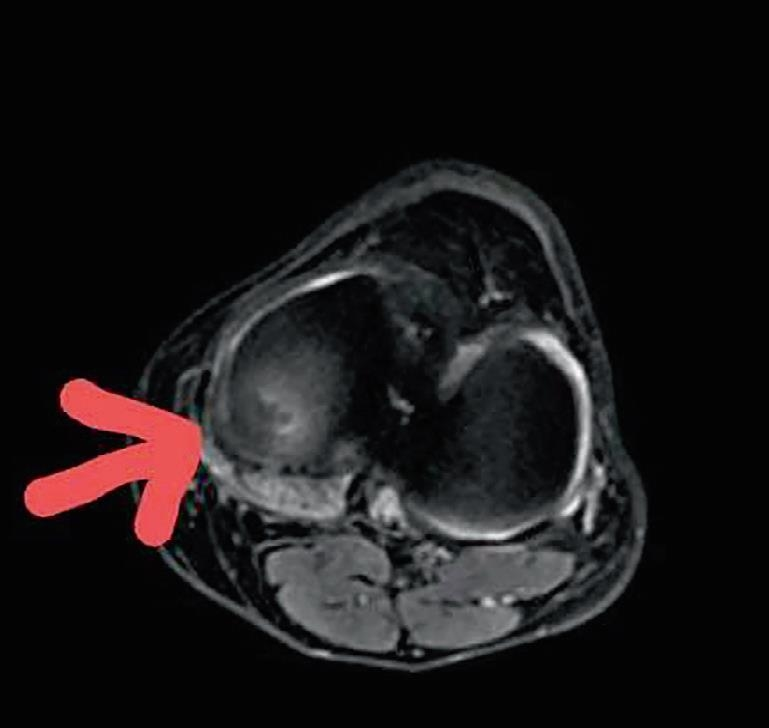
(b)

#### 2.4.1. Differential Diagnosis

The differential diagnosis for osteochondral lesions in a middle‐aged patient includes spontaneous osteonecrosis of the knee (SONK), posttraumatic osteochondral fracture, degenerative chondral lesions, and OCD. SONK typically presents with acute onset of severe pain, affects the weight‐bearing surface of the medial femoral condyle, and demonstrates characteristic subchondral crescent sign with condylar flattening on MRI. In contrast, our patient exhibited a gradual symptom onset over 6 months, and the lesion was located on the lateral aspect of the medial femoral condyle—the classic predilection site for OCD. Posttraumatic osteochondral fractures typically demonstrate a clear history of acute injury and acute bone marrow edema patterns, neither of which were present in our case. Degenerative chondral lesions are usually associated with diffuse cartilage loss and widespread osteoarthritic changes; however, our patient demonstrated a focal, well‐circumscribed lesion without significant generalized chondropathy. The MRI findings of a clearly demarcated osteochondral fragment with surrounding bone marrow edema at the characteristic lateral aspect of the medial femoral condyle, combined with arthroscopic confirmation of an unstable osteochondral fragment consistent with Stage 4B OCD, supported the definitive diagnosis of OCD.

#### 2.4.2. Diagnostic Workup Rationale

Computed tomography (CT) was not performed as part of the preoperative workup. At our institution, the combination of plain radiography and MRI is considered sufficient for preoperative assessment prior to arthroscopic intervention in osteochondral lesions, as MRI provides superior soft tissue characterization and is the gold standard for evaluating cartilage integrity, subchondral bone status, and lesion stability. Although CT may offer enhanced visualization of osseous detail and calcification morphology, it was deemed unnecessary given the adequate diagnostic information obtained from MRI. Similarly, histopathological examination of the excised calcified body was not pursued. The imaging characteristics were consistent with a benign meniscal calcification/ossicle, and the clinical presentation did not suggest underlying malignancy or systemic calcification disorders requiring tissue diagnosis. We acknowledge these decisions represent limitations of this case report; however, arthroscopic examination served as the definitive diagnostic and therapeutic modality, providing direct visualization and confirmation of both the OCD lesion characteristics and the meniscal calcification.

### 2.5. Therapeutic Intervention

Based on these findings, arthroscopic surgery was performed. Intraoperatively, the calcified body attached to the posterior horn of the medial meniscus was identified in the posteromedial compartment, adjacent to the OCD lesion on the weight‐bearing surface of the medial femoral condyle, and arthroscopically excised (Figure 5; Video S1 demonstrates the surgical procedure including identification of the OCD lesion, excision of the meniscal calcified body, and microfracture technique). Subsequently, the unstable Stage 4B OCD lesion was debrided. Given that the patient was not scheduled for arthroplasty at the time of surgery, a joint‐preserving approach was preferred. Considering the size and location of the defect and the absence of significant diffuse chondral loss, a microfracture technique was applied to promote cartilage healing.

Figure 5(a) Arthroscopic view of osteochondritis dissecans lesion on the lateral aspect of the medial femoral condyle, demonstrating the unstable osteochondral fragment. (b) Arthroscopic view of a calcified loose body (meniscus stone) within the posterior horn of the medial meniscus, located in close proximity to the OCD lesion. (c) Gross specimen of a calcified meniscal loose body following arthroscopic excision.

**Figure:**
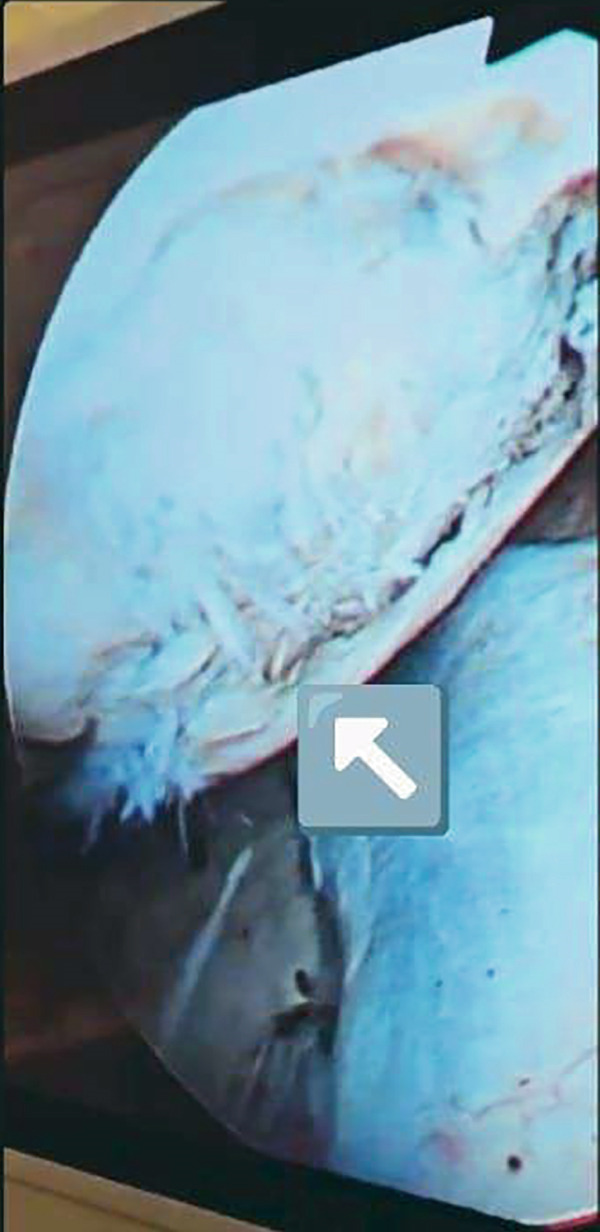
(a)

**Figure:**
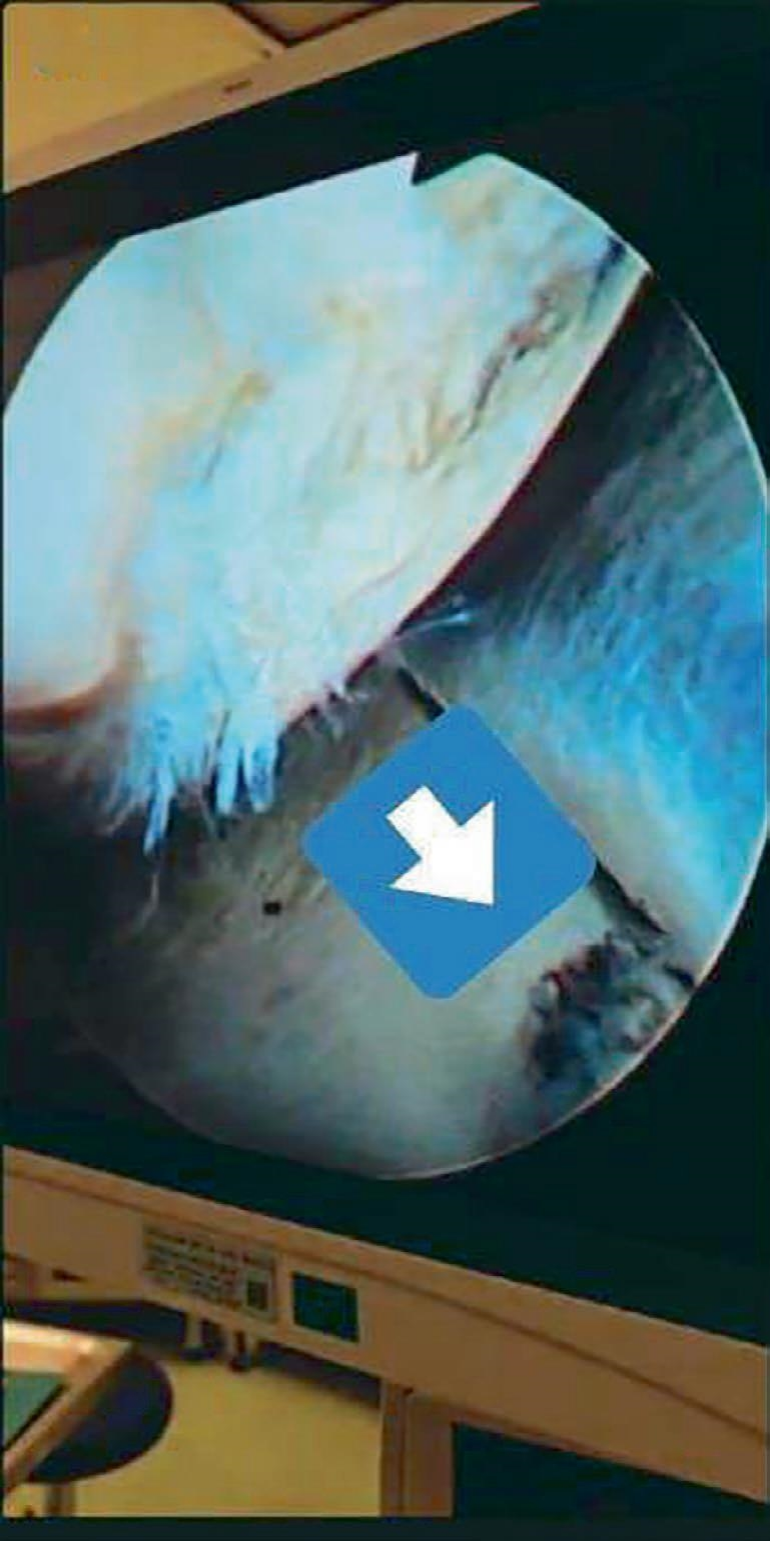
(b)

**Figure:**
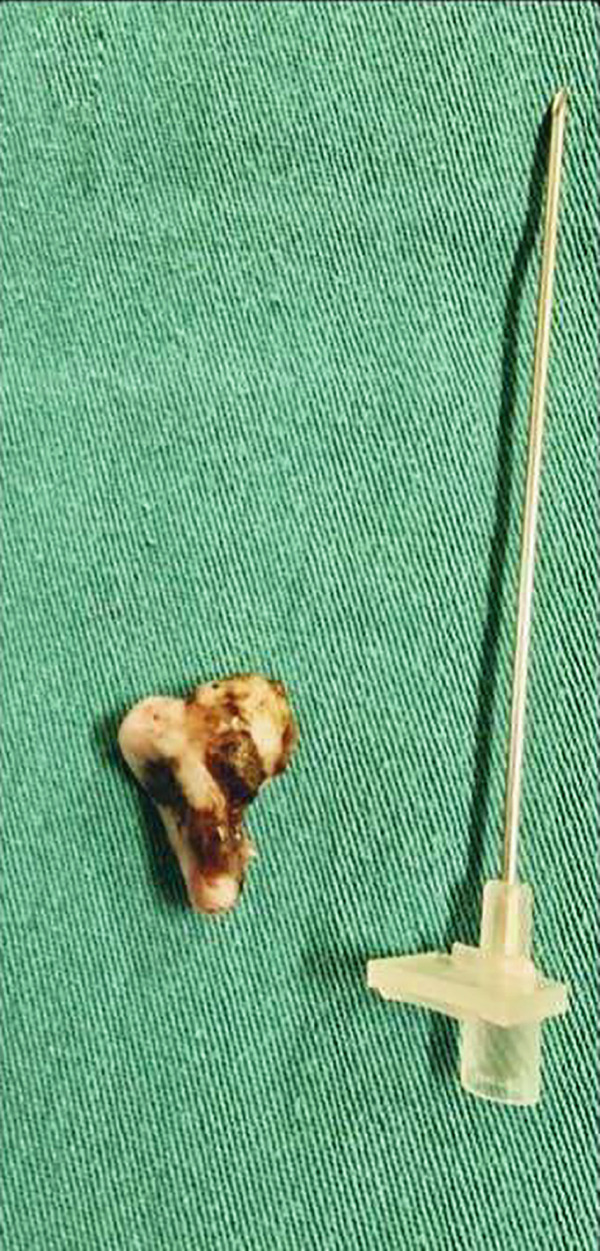
(c)

#### 2.5.1. Surgical Technique Details

The procedure was performed under spinal anesthesia with the patient in a supine position and a thigh tourniquet inflated to 300 mmHg. Standard anterolateral and anteromedial portals were established for initial joint inspection and instrumentation. For accessing the posteromedial compartment containing the calcified body, a posteromedial portal was created using an inside‐out technique under direct arthroscopic visualization. The portal was established approximately 1 cm above the joint line, posterior to the medial collateral ligament, with careful attention to avoid the saphenous nerve and vein. A switching stick was introduced through the intercondylar notch to tent the posteromedial capsule, and the portal was created under direct visualization using a spinal needle followed by a #11 blade. Instruments utilized included a 4.0‐mm arthroscope (30° lens), arthroscopic grasper, curette, motorized shaver (4.5‐mm aggressive blade), and microfracture awls. The calcified body was carefully dissected from its attachment to the posterior horn of the medial meniscus using a combination of radiofrequency ablation and mechanical debridement. Special care was taken to preserve the remaining meniscal tissue while ensuring complete excision of the calcified mass. The OCD lesion was then addressed through the standard portals, with debridement of unstable cartilage and subchondral bone preparation followed by microfracture using 45° and 90° awls spaced 3–4 mm apart to achieve appropriate marrow element extravasation.

### 2.6. Follow‐Up and Outcomes

The postoperative course was uneventful. The patient was mobilized with partial weight bearing for 6 weeks, followed by a structured physiotherapy program. At the 6‐month follow‐up, the patient reported significant improvement with reduction of pain, resolution of locking, and decreased swelling. Clinical outcome measures demonstrated substantial improvement: VAS pain score decreased from 7 preoperatively to 3 at 6 months postoperatively, and KOOS improved from 15 preoperatively to 75 at 6 months postoperatively. Range of motion improved from 0°–110° preoperatively to 0°–125° at 6 months postoperatively (Table 1). Follow‐up imaging was performed with plain radiographs rather than MRI. This decision was based on routine clinical practice for elderly patients who are potential candidates for future arthroplasty, where cost‐effectiveness and clinical indication guide imaging selection. Radiographic follow‐up confirmed stability of the OCD lesion with no recurrence of intra‐articular loose bodies.

**Table 1:** Summarizes the clinical outcomes at baseline, interim (6 weeks), and 6‐month follow‐up.

Parameter	Baseline (preoperative)	6 Weeks postoperative	6 Months postoperative
VAS pain score	7/10	4/10	3/10
KOOS total	15	52	75
Range of motion	0°–110°	0°–115°	0°–125°
Weight‐bearing status	Full, with pain	Partial	Full, minimal pain
Joint effusion	Mild	Minimal	Absent
Mechanical symptoms	Present (locking)	Absent	Absent
Activity level	Limited	Restricted	Near‐normal

### 2.7. Patient Perspective

Prior to surgery, the patient reported significant impairment in daily activities, stating: “The unpredictable locking episodes made me fearful of walking, especially on stairs or uneven surfaces. I had to give up my morning walks and could not kneel for prayer, which was emotionally distressing.” Following arthroscopic intervention, the patient noted substantial improvement: “Within three months, I could walk without fear of my knee giving way. The constant aching has reduced considerably, and I have returned to my daily walking routine. I can now perform my prayers comfortably and climb stairs with confidence.” At the 6‐month follow‐up, the patient expressed high satisfaction with the surgical outcome and reported near‐complete restoration of his premorbid functional status.

## 3. Discussion

OCD is a joint disorder characterized by focal subchondral lesions that initiate with impaired ossification at the osteochondral junction, progressing through transitional discontinuity, and potentially culminating in subchondral bone necrosis and potential detachment of an osteochondral fragment, most commonly affecting the knee joint. Current understanding suggests that necrosis represents a late‐stage finding rather than the primary pathological event, with the initial insult occurring at the subchondral osteochondral interface [4]. The etiology of OCD is multifactorial, involving repetitive microtrauma, vascular insufficiency, genetic predisposition, and abnormal biomechanical stresses [8]. In recent years, increasing attention has been paid to the role of localized mechanical stress in the development and progression of OCD lesions.

Although OCD predominantly occurs in adolescents and young adults, its occurrence in older patients, such as our 56‐year‐old patient, warrants consideration of alternative diagnoses including osteochondral fracture from unrecognized trauma or SONK. However, in our case, the characteristic imaging features, lesion location on the lateral aspect of the medial femoral condyle (the classic OCD site), and clinical presentation were consistent with OCD rather than SONK, which typically presents with acute onset pain and affects the weight‐bearing portion of the medial femoral condyle. The possibility of a chronic, silent traumatic etiology cannot be entirely excluded; however, the patient′s clinical history and lesion characteristics favor a diagnosis of adult‐onset OCD, potentially representing late presentation of juvenile OCD or de novo adult OCD accelerated by the mechanical effects of the adjacent meniscal calcification.

Intra‐articular loose bodies are well‐known sources of chronic mechanical irritation within the joint space. These bodies typically arise from trauma, osteochondral fractures, degenerative joint diseases, or synovial chondromatosis, and can accelerate cartilage degeneration and osteochondral damage [9]. Although free‐floating loose bodies are relatively common in orthopedic practice, calcified bodies fixed to intra‐articular structures, such as the meniscus, are exceedingly rare [10].

A review of the literature reveals only a few reports describing meniscal ossicles or calcified bodies originating from degenerative changes of the meniscus. Vangrinsven et al. [6] reported a case of a meniscal ossicle mimicking a loose body, highlighting diagnostic challenges. However, to date, no clear association has been reported between a meniscal‐fixed calcified body and the development of OCD.

The relationship between OCD and meniscal calcification in our case merits careful consideration. Although the coexistence of these two pathologies could theoretically be coincidental, several factors suggest a potential pathophysiological relationship: (1) the anatomical proximity of the calcified body to the OCD lesion, (2) the location of the calcified body within the projection area of the OCD lesion, and (3) the biomechanical plausibility that a fixed calcified body could generate repetitive focal stress on the overlying osteochondral unit during joint motion. Nevertheless, we acknowledge that a causal relationship cannot be definitively established from a single case observation.

In the present case, the presence of an irregularly contoured calcified body attached to the posterior horn of the medial meniscus, located in the projection area of a Stage 4B OCD lesion, suggests that chronic mechanical pressure may have contributed to the initiation or progression of the osteochondral defect. This observation supports the hypothesis proposed by Gans et al. [11], who emphasized that persistent intra‐articular mechanical factors could destabilize osteochondral units in susceptible joints.

Synovial chondromatosis has occasionally been reported in association with OCD, where multiple loose bodies exacerbate joint degeneration. Filho et al. [12] presented such a case, noting the potential for loose bodies to accelerate OCD progression. Unlike synovial chondromatosis, our case involved a single, fixed calcified body, marking a unique clinical presentation.

Treatment of Stage 4 OCD lesions generally necessitates surgical intervention due to lesion instability [13]. Options include fragment fixation, osteochondral autograft transplantation (OATS), autologous chondrocyte implantation (ACI), or palliative approaches such as debridement and microfracture [14, 15]. Although microfracture is traditionally recommended for smaller, contained defects, it remains a viable option for moderate‐sized lesions in appropriately selected patients. The decision to perform microfracture in our patient, despite his age, was based on several considerations: (1) the patient was not a planned arthroplasty candidate at the time of surgery, (2) the absence of diffuse chondral loss or advanced osteoarthritic changes, (3) the contained nature of the defect, and (4) patient preference for a joint‐preserving procedure. Steadman et al. [16] reported favorable outcomes using microfracture in OCD cases under specific conditions. In our patient, considering the lesion size (~1.5 cm), location, and patient‐specific factors, microfracture was chosen as a less invasive and cost‐effective treatment, resulting in successful short‐term outcomes.

This case highlights a rare but clinically significant scenario where a meniscal‐fixed calcified body may act as a mechanical trigger in OCD pathogenesis. It emphasizes the importance of comprehensive imaging in patients presenting with mechanical symptoms and osteochondral lesions, as identifying such contributing factors can significantly influence surgical planning and prognosis.

Further research is warranted to better understand the pathophysiological relationship between fixed intra‐articular calcifications and OCD development, and to establish optimal management strategies for similar cases.

### 3.1. Learning Points

The learning points of this study are as follows:

1. Adult‐onset OCD requires careful differentiation from SONK: Lesion location on the lateral aspect of the medial femoral condyle (classic OCD site) versus the weight‐bearing surface (typical SONK location), gradual versus acute symptom onset, and MRI characteristics help distinguish these entities.
2. Meniscal calcifications, while often incidental findings, may contribute to mechanical symptoms and should be evaluated in the context of associated intra‐articular pathology: Their proximity to osteochondral lesions may suggest a pathophysiological relationship warranting surgical consideration.
3. MRI remains sufficient for preoperative planning in most osteochondral lesions: Although CT may provide additional osseous detail, MRI adequately characterizes lesion stability, cartilage integrity, and associated soft tissue abnormalities, with arthroscopy serving as the definitive diagnostic and therapeutic modality.
4. Joint‐preserving microfracture remains a viable option for selected older patients not planned for arthroplasty: In the absence of diffuse chondropathy and with contained lesions, microfracture can provide satisfactory short‐ to medium‐term outcomes while preserving future surgical options.
5. Patient‐reported outcome measures (VAS and KOOS) are essential for documenting functional improvement and should guide clinical decision‐making during follow‐up: These validated instruments provide objective evidence of treatment efficacy and patient satisfaction.

## 4. Conclusion

This case presents a rare association between OCD of the medial femoral condyle and a meniscal‐fixed calcified body, highlighting the potential role of chronic localized mechanical stress in OCD pathogenesis. Although a coincidental association cannot be excluded, the anatomical and biomechanical relationship between these findings suggests a possible pathophysiological link. Comprehensive imaging is crucial to detect such contributing factors, which may alter surgical decision‐making. Arthroscopic removal combined with microfracture provided a favorable clinical outcome at 6 months, with significant improvement in VAS pain score (7–3) and KOOS (15–75). Range of motion improved from 0°–110° to 0°–125°, and the patient reported near‐complete restoration of daily activities. Further studies are needed to clarify the impact of intra‐articular calcifications on OCD progression and to establish optimal treatment strategies.

## Author Contributions

Ekrem Özdemir: conceptualization, data collection, surgical procedure, manuscript drafting, and revision. Nasuhi Altay: supervision, critical review, and final approval of the manuscript.

## Funding

No funding was received for this manuscript.

## Disclosure

Both authors have read and approved the final manuscript.

## Ethics Statement

Written informed consent was obtained from the patient for publication of this case report and accompanying images, including clinical photographs, radiographic images, MRI scans, and surgical video. The patient was informed about the nature of the publication, the potential audience, and the measures taken to protect patient confidentiality. The signed consent form is securely stored at Erzurum City Hospital, Department of Orthopedics and Traumatology, in accordance with institutional data protection policies and applicable regulations. This case report was conducted in compliance with the ethical principles outlined in the Declaration of Helsinki. As this is a retrospective case report without experimental intervention, formal ethics committee approval was not required per institutional guidelines; however, patient privacy and confidentiality were strictly maintained throughout the preparation of this manuscript.

## Conflicts of Interest

The authors declare no conflicts of interest.

## Supporting Information

Additional supporting information can be found online in the Supporting Information section.

## Supporting information


**Supporting Information 1** Video S1: Arthroscopic video demonstrating the surgical procedure including: identification of the Stage 4B osteochondritis dissecans lesion on the medial femoral condyle, visualization and excision of the calcified meniscal body (meniscus stone) attached to the medial meniscus, debridement of the unstable OCD fragment, and application of the microfracture technique to the prepared lesion bed.

**Supporting Information 2** CARE checklist.
